# Child mortality in England after national lockdowns for COVID-19: An analysis of childhood deaths, 2019–2023

**DOI:** 10.1371/journal.pmed.1004417

**Published:** 2025-01-23

**Authors:** David Odd, Sylvia Stoianova, Tom Williams, Peter Fleming, Karen Luyt

**Affiliations:** 1 Division of Population Medicine, School of Medicine, Cardiff University, Cardiff, United Kingdom; 2 National Child Mortality Database, Bristol Medical School, St Michael’s Hospital, University of Bristol, Bristol, United Kingdom; 3 Centre for Academic Child Health, Population Health Sciences, Bristol Medical School, University of Bristol, Bristol, United Kingdom; London School of Hygiene and Tropical Medicine, UNITED KINGDOM OF GREAT BRITAIN AND NORTHERN IRELAND

## Abstract

**Background:**

During the COVID-19 pandemic children and young people (CYP) mortality in England reduced to the lowest on record, but it is unclear if the mechanisms which facilitated a reduction in mortality had a longer lasting impact, and what impact the pandemic, and its social restrictions, have had on deaths with longer latencies (e.g., malignancies). The aim of this analysis was to quantify the relative rate, and causes, of childhood deaths in England, before, during, and after national lockdowns for COVID-19 and its social changes.

**Methods and findings:**

Deaths of all children (occurring before their 18th birthday) occurring from April 2019 until March 2023 in England were identified. Data were collated by the National Child Mortality Database. Study population size and the underlying population profile was derived from 2021 Office of National Statistics census data Mortality for each analysis year was calculated per 1,000,000 person years. Poisson regression was used to test for an overall trend across the time period and tested if trends differed between April 2019 to March 2021 (Period 1)) and April 2021 to March 2023 (Period 2: after lockdown restrictions). This was then repeated for each category of death and demographic group. Twelve thousand eight hundred twenty-eight deaths were included in the analysis. Around 59.4% of deaths occurred under 1 year of age, 57.0% were male, and 63.9% were of white ethnicity. Mortality rate (per 1,000,000 CYP per year) dropped from 274.2 (95% CI 264.8–283.8) in 2019−2020, to 242.2 (95% CI 233.4–251.2) in 2020−2021, increasing to 296.1 (95% CI 286.3–306.1) in 2022−2023. Overall, death rate reduced across Period 1 (Incidence rate ratio (IRR) 0.96 (95% CI 0.92–0.99)) and then increased across Period 2 (IRR 1.12 (95% CI 1.08–1.16)), and this pattern was also seen for death by Infection and Underlying Disease. In contrast, rate of death after Intrapartum events increased across the first period, followed by a decrease in rate in the second (Period 1 IRR 1.15 (95% CI 1.00–1.34)) versus Period 2 (IRR 0.78 (95% CI 0.68–0.91), *p*_difference_ = 0.004). Rates of death from preterm birth, trauma and sudden unexpected deaths in infancy and childhood (SUDIC), increased across the entire 4-year-study period (preterm birth, IRR 1.03 (95% CI 1.00–1.07); trauma IRR 1.12 (95% CI 1.06–1.20); SUDIC IRR 1.09 (95% CI 1.04–1.13)), and there was no change in the rate of death from Malignancy (IRR 1.01 (95% CI 0.95–1.06)). Repeating the analysis, split by child characteristics, suggested that mortality initially dropped and subsequently rose for children between 1 and 4 years old (Period 1 RR 0.85 (95% CI 0.76–0.94) versus Period 2 IRR 1.31 (95% CI 1.19–1.43), *p*_difference_ < 0.001. For Asian, black and Other ethnic groups, we observed increased rates of deaths in the period 2021−2023, and a significant change in trajectory of death rates between Periods 1 and 2 (Asian (Period 1 IRR 0.93 (95% CI 0.86–1.01) versus Period 2 IRR 1.28 (95% CI 1.18–1.38), *p*_difference_ < 0.001); black (Period 1 IRR 0.97 (95% CI 0.85–1.10) versus Period 2 IRR 1.27 (95% CI 1.14–1.42), *p*_difference_ = 0.012); Other (Period 1 IRR 0.84 (95% CI 0.68–1.04) versus Period 2 IRR 1.45 (95% CI 1.20–1.75), *p*_difference_ = 0.003). Similar results were observed in CYP in the most deprived areas (Period 1 IRR 0.95 (95% CI 0.89–1.01) versus Period 2 IRR 1.18 (95% CI 1.12–1.25), *p*_difference_ < 0.001). There was no change in the trajectory of death rates for children from white (*p* = 0.601) or mixed (*p* = 0.823) ethnic backgrounds, or those in the least deprived areas (*p* = 0.832), between Periods 1 and 2; with evidence of a rise across the whole study period for children from white backgrounds (IRR 1.05 (95% CI 1.03–1.07), *p* < 0.001) and those in the least deprived areas (IRR 1.06 (95% CI 1.01–1.10), *p* < 0.001). Limitations include that the population at risk was estimated at a mid-point of the study, and changes may have biased our estimates. In particular, absolute rates should be interpreted with caution. In addition, child death in England is rare, which may further limit interpretation; particularly in the stratified analyses.

**Conclusions:**

In this study, overall child mortality in England after the national lockdowns was higher than before them. We observed different temporal profiles across the different causes of death, with reassuring trends in deaths from Intrapartum deaths after lockdowns were lifted. However, for all other causes of death, rates are either static, or increasing. In addition, the relative rate of dying for children from non-white backgrounds, compared to white children, is now higher than before or during the lockdowns.

## Author summary

**Why was this study done?:**

- We know that, during the national lockdowns initiated due to the COVID-19 pandemic, the number of children dying in England was lower than seen prior to the pandemic.
- The reduction in deaths appeared to be across most age groups, regions and areas of England, and in both boys and girls.
- The number of deaths increased once England returned to pre-pandemic rules, but we don’t know what happened to the rate of death after this, and if rate of death varied according to child characteristics such as age, sex and ethnicity.

**What did the researchers do and find?:**

- We identified all those children in England who died between April 2019 and March 2023 and calculated what the rate of death was for each year, for each group of children (e.g., infants or older children) and what they appeared to die of.
- Using a mathematical model we then tested if the rate of death was going up or down across the 4 years, and if the trend seen was different for the first two years (before and during the national lockdowns) compared to the second two (after the national lockdowns).
- Overall, children were less likely to die during the pandemic (April 2020–March 2021) than at any other time, with 377 fewer deaths than expected from the previous year. The number of deaths in the following year was similar to before the pandemic, but in 2022−2023, there were 258 more deaths than expected from the pre-pandemic period.
- While mortality in the most deprived areas, and in children of non-white ethnicities, did not drop during the pandemic, they demonstrated significant increases in the post-lockdown period, making the relative rate of dying for children from non-white backgrounds, compared to white children, higher than before or during the pandemic.

**What do these findings mean?:**

- Overall, child mortality after the national lockdowns was higher than during, or preceding them.
- While deaths in most categories appear to have increased or remained the same, intrapartum Deaths appeared to increase during the national lockdowns, but then reduced afterwards.
- As the number of children in the population is based on census data, this work may not perfectly reflect the actual number of children in England and so the absolute rates of death have some uncertainty around them. Equally, the category of death (e.g., infection) is often based on early information and might change as more information is known through the Child Death Review process.

## Abstract 3

David Odd and colleagues analyse rates and causes of childhood deaths in England before, during, and after the COVID pandemic, and assess temporal trends for categories of death and demographic groups.

## Introduction

The COVID-19 pandemic probably caused over 14 million deaths worldwide [1], and was associated with broad and profound changes in how people lived and worked. However, the number of deaths from COVID in children and young people (CYP) was low; around 1% of CYP deaths during the pandemic were probably caused by the COVID-19 virus [2,3]. In contrast, overall mortality for CYP reduced during the first year of the pandemic (2020), associated with the periods of broad legal restrictions, including closing of schools, shops and recreational activity (“lockdowns”), put in place to reduce the spread of COVID-19 [4,5], with dramatic reductions in infections and deaths from underlying conditions [6]. However, since the end of the national lockdowns, the rate of child deaths in England returned to levels comparable to the pre-pandemic period [6], leaving a cohort of around 350 more children alive at that point due to reductions in mortality over the pandemic years. The reductions seen were similar across age groups, regions of England, sex and socio-economic deprivation [7]. Since then, England, like most countries, has rescinded its COVID policies and legislation, and returned to pre-pandemic regulations and operation [8]. It is unclear if the public health mechanisms, associated with the reduction in mortality in vulnerable groups [6], has had a longer lasting impact, and what impact the pandemic, and its social restrictions, have had on deaths with longer latencies (e.g., malignancies), first developing during the highest period of disruption when later diagnosis, or treatment, may have occurred. Equally, while overall child mortality fell, concerns remained in some categories (e.g., suicide [9]) and urban communities, where lasting societal and behavioural changes may have occurred.

The aim of this analysis was to quantify the relative rate of childhood deaths, across the whole of England, before, during, and after the COVID-19 national lockdowns and its social changes, and identify any changes that may have occurred, and the patterns of mortality, during this period.

## Methods

### Data, study design and population

In England, all deaths of children (0–17 years old) are reviewed by 58 Child Death Overview Panels (CDOPs), and data, from the 1st of April 2019, collated by the National Child Mortality Database (NCMD) [10]. Analysis was performed on data received by NCMD by the 14th September 2023. The study population was derived from 2021 Office of National Statistics (ONS) census data [11], and includes measures of ethnicity, sex and age.

### Outcome

Deaths of all CYP, who died before their 18th birthday, and had been born at, or after, 22 weeks of gestation, occurring from 1st April 2019 until 31st of March 2023 were identified. In this work, similar to our previous work [5], in order to obtain a provisional category of death, all child deaths reported to NCMD were coded by three independent coders (all paediatricians) to identify the likely category of the cause of death. All coders recorded a provisional category of death (see below) or that there was insufficient information provided. For each death, if two or more coders agreed on a category this was taken as the most likely category and where no two coders agreed, the category highest in the following hierarchy was used (based on categorisation used by CDOPs [12], in order of priority: trauma, malignancy, underlying medical condition, intrapartum event, preterm birth, infection, sudden unexpected death in infant and childhood (SUDIC).

### Covariates

In the initial notification to NCMD (by statute this is within 48 h of the death), the CDOPs report baseline characteristics of the child; from which the following data were derived: sex of individual (female, male, other (including not known)), age at death, ethnicity (directly derived data from NHS professionals and data records during the CDOP process), and the child’s home postcode. The postcode was then mapped to a geographic measure, the middle layer super output area, which is derived at granularity of around 7,200 people [13]. This code was used to identify the CYP’s region of England, the rural/urban status, and a local measure of deprivation: the index of multiple deprivation (IMD). The IMD is calculated using seven main domains (income, employment, education, health, crime, access to housing and services, and living environment). The population split into 10 equal sized (by people) deciles 1 being most deprived and 10 least deprived [14]. Ethnic group was derived from NHS data, and is parent reported at source, and classified using ONS guidelines; Asian or Asian British (Bangladeshi, Chinese, Indian, Pakistani, any other Asian background), black or black British (African, Caribbean, any other black background), multi-racial (white and Asian, white and black African, white and black Caribbean, any other multi-racial background), white or white British (British, Irish, any other white background, Gypsy or Irish traveller), and other (Arab, any other ethnic group).

### Statistical analysis

Deaths of children occurring from 1st April 2019 until 31st March 2023, born at, or after 22 completed weeks of gestation, were identified and divided into four 12-month periods; 1st April to 31st March for each year; starting in April 2019 with the start of NCMD’s data collection. A priori, the first two years of the period (April 2019–March 2021) were classified as Period 1, while the 2 years after the peak of the pandemic over the winter of 2021/2022, and the lifting of the national lockdowns, was considered Period 2 (April 2021–March 2023). Statistical analysis was planned a priori in June 2023.

Initially, the demographics for childhood deaths, split by the year were compared from reported data. Demographic comparisons were made using the *χ*^2^ test. Rates of overall death, and for each category, were derived for each year. Next, the underlying population size and characteristics were derived from ONS 2021 Census data. The demographics of children dying were compared across years using the *χ*^2^ statistics, and the rate of death, for each analysis year was calculated per 1,000,000 children per year. A random effects Poisson regression model (using month of death as the group variable) was used to test for an overall trend across the 4 years, along with the incidence rate ratio (IRR) of death per year. For all analyses data was collapsed down to events per month and the rate of death seen that month, and the trends, generated using the Poisson model. Units of time were in years (e.g., with decimal points indicating the month) so that the final analysis/trends are reported for change per year for ease of interpretation. Where a covariate was missing (e.g., ethnicity), the deaths were removed from that analysis only, and number for each analysis are shown. For the graphical outputs (i.e., not the analyses) monthly estimates are as smoothed to give the reader a better idea of changes across time and to remove the risk of identifiable data being reported. Smoothing was done using a Gaussian function over a 5-month window, to maintain the weight of key monthly events, but obtain the above impacts. Next, a linear spline model was fitted allowing different trends in Periods 1 and 2. A *p*-value was derived to test for a change of trend at this mid-point by comparing the trend predicted in Period 2, from that predicted in Period 1 (the counterfactual). This was repeated for each cause of death and the child’s characteristics (above) and interaction *p*-values derived as evidence that the profile may differ by the stratified characteristic (e.g., sex).

To quantify changes in health inequalities across the 4 years, the relative rate of death for children living in the more deprived half of England (Deciles 1–5), was compared to those in the less deprived half (Deciles 6–10), for each of the four years of data, alongside tests for trend across the two a priori time periods (as above). This analysis was then repeated, comparing mortality for children from non-white ethnicities, and each specific ethnic group, with those from white backgrounds.

After suggestions from the peer-review process, the main analysis of all deaths, and the categories of death, after review, was repeated, split by the age category of the children and, then restricted after removing the deaths of children born at 23 and 24 weeks of gestation. The number of likely additional deaths seen, for each year, compared to the first (and pre-lockdown) year of 2019−2020 were derived. Estimates were derived using a generalised linear model (Poisson family) and repeated for each provisional category of death. This work was reviewed by the Chair of the Central Bristol NHS Research Ethics Committee who confirmed that NHS ethical approval, including obtaining individual consent, was not required. Data are presented as number (%), rate per 1,000,000 children per year (95% CI), IRR (95% CI), or excess deaths (number) with 95% CI. All tests were two-sided. This study is reported as per the Reporting of Studies Conducted using Observational Routinely-Collected Data (RECORD) guideline (S1 RECORD Checklist). Analysis was performed using Stata Version 17.

### Patient and public involvement

Parent and public involvement guided the design and setting up of the NCMD at establishment and real-time child mortality surveillance system at the beginning of the COVID-19 pandemic.

### Ethics approval and consent to participate

The NCMD legal basis to collect confidential and personal level data under the Common Law Duty of Confidentiality has been established through the Children Act 2004 Sections M-N, Working Together to Safeguard Children 2018 [15] and associated Child Death Review Statutory and Operational Guidance [10]. The NCMD legal basis to collect personal data under the General Data Protection Regulation (GDPR) without consent is defined by GDPR Article 6 (e) Public task and 9 (h) Health or social care (with a basis in law).

## Results

Over the 4 years, a total of 13,743 deaths were reported to NCMD, of which 915 were excluded for birth before 22 weeks gestation, leaving 12,828 deaths; 3,229 deaths in 2019−2020, 2,852 in 2020−2021, 3,260 in 2021−2022 and 3,487 in 2022/2023 (Table 1). Over half (59.4% (7,622/12,828) of deaths were of children less than 1 year of age, 57.0% (7,275/12,774) were male, 63.9% (7,758/12,142) were of white ethnicity and London had more deaths than any other region (17.1% (2,192/12,828).

**Table 1:** Characteristics of the populations of child deaths reported to NCMD in England between April 2019 and March 2023.

Measure	*N*	Child deaths reported—number (%)				*P* (*χ*^2^)
		2019/2020	2020/2021	2021/2022	2022/2023	
All deaths	12,828	3,229	2,852	3,260	3,487	–
Age of death	12,828					<0.001
<1 years	7,622	1,952 (60.5%)	1,772 (62.1%)	1,943 (59.6%)	1,955 (56.1%)	
1–4 years	1,475	397 (12.3%)	270 (9.5%)	367 (11.3%)	441 (12.7%)	
5–15 years	2,643	629 (19.5%)	563 (19.7%)	662 (20.3%)	789 (22.6%)	
15–17 years	1,088	251 (7.8%)	247 (8.7%)	288 (8.8%)	302 (8.7%)	
Sex	12,774					0.196
Female	5,499	1,417 (44.1%)	1,236 (43.7%)	1,397 (42.9%0	1,449 (41.7%)	
Male	7,275	1,795 (55.9%)	1,594 (56.3%)	1,857 (57.1%)	2,029 (58.3%)	
Area of residence	12,782					0.036
Rural	1,450	391 (12.2%)	284 (10.0%)	382 (11.8%)	393 (11.3%)	
Urban (London)	2,187	570 (7.8%)	516 (18.2%)	521 (16.0%)	580 (16.7%)	
Urban (not London)	9,145	2,251 (70.1%)	2,043 (71.9%)	2,349 (72.2%)	2,502 (72.0%)	
Ethnicity	12,142					<0.001
Asian or British Asian	2,242	549 (18.7%)	442 (16.9%)	572 (17.8%)	679 (20.2%)	
Black or British black	1,039	236 (8.0%)	229 (8.8%)	249 (7.7%)	325 (9.7%)	
Mixed	742	190 (6.5%)	147 (5.6%)	210 (6.5%)	195 (5.8%)	
Other^a^	361	90 (3.1%)	63 (2.4%)	86 (2.7%)	133 (3.6%)	
White	7,758	1,876 (63.8%)	1,737 (66.4%)	2,099 (65.3%)	2,046 (60.8%)	
Region of CDOP	12,828					0.803
East Midlands	1,054	266 (8.2%)	232 (8.1%)	275 (8.4%)	281 (8.1%)	
East of England	1,233	329 (10.2%)	261 (9.2%)	318 (9.8%)	325 (9.3%)	
London	2,192	573 (17.8%)	516 (18.1%)	521 (16.0%)	582 (16.7%)	
North East	633	147 (4.6%)	127 (4.5%)	175 (5.4%)	184 (5.3%)	
North West	1,903	460 (14.3%)	422 (14.8%)	490 (15.0%)	531 (15.2%)	
South East	1,706	442 (13.7%)	367 (12.9%)	443 (13.6%)	454 (13.0%)	
South West	947	241 (7.5%)	226 (7.9%)	235 (7.2%)	245 (7.0%)	
West Midlands	1,721	416 (12.9%)	383 (13.4%)	442 (14.6%)	480 (13.8%)	
Yorkshire and the Humber	1,439	355 (11.0%0	318 (11.2%)	361 (11.1%)	405 (11.6%)	
Deprivation decile	12,753					0.252
1/2 (Most deprived)	4,377	1,077 (33.6%)	963 (33.9%)	1,088 (33.5%)	1,249 (35.1%)	
3/4	2,904	730 (22.8%)	646 (22.8%)	729 (22.5%)	799 (23.1%)	
5/6	2,236	593 (18.5%)	510 (18.0%)	558 (17.2%)	575 (16.6%)	
7/8	1,760	457 (14.3%)	383 (13.5%)	466 (14.4%)	454 (13.1%)	
9/10 (least deprived)	1,476	350 (10.9%)	335 (11.8%)	403 (12.4%)	388 (11.2%)	

*P*-values derived from *χ*^2^ testing.

^a^Other ethnic group (Arab, Any other ethnic group).

Overall rate of death (per 1,000,000 children per year) dropped from 274.2 (95% CI 264.8–283.8) in 2019−2020 to 242.2 (95% CI 233.4–251.2) in 2020−2021, then increased to 276.8 (95% CI 267.4–286.5) in 2021−2022, and then to 296.1 (95% CI 286.3–306.1) in 2022−2023 (*p*_trend_ < 0.001) (Tables 2 and S1). Across the whole time period, there was evidence of an increase in the rate of death from preterm birth (*p*_trend_ = 0.046), infection (*p*_trend_ < 0.001), trauma (*p*_trend_ < 0.001), SUDIC (*p*_trend_ < 0.001) and underlying disease (*p*_trend_ = 0.006) (Table 3). There was no evidence for an overall trend in the rate of deaths from malignancy (*p*_trend_ = 0.831), intrapartum events (*p*_trend_ = 0.135), illicit drug use (*p*_trend_ = 0.363) or suicide (*p*_trend_ = 0.849) over the study period (Table 3).

**Table 2:** Rate (per 1,000,000 children per year), and relative rate and excess deaths (compared to 2019−2020), by year of death, stratified by cause of death (total population estimate = 11,777,798).

Measure	*N*	Rate (per 1,000,000 children per year)			
		2019–2020	2020–2021	2021–2022	2022–2023
**Rate of death per 1,000,000 children**					
All deaths	12,828	274.2 (264.8 to 283.8)	242.2 (233.4 to 251.2)	276.8 (267.4 to 286.5)	296.1 (286.3 to 306.1)
*Death by cause*					
Malignancy	1,041	22.0 (19.4 to 24.8)	22.4 (19.8 to 25.3)	21.2 (18.7 to 24.0)	22.8 (20.1 to 25.7)
Preterm birth	2,860	59.3 (55.0 to 63.9)	56.1 (51.9 to 60.6)	64.3 (59.8 to 69.0)	63.1 (58.6 to 67.8)
Intrapartum event	686	14.2 (12.1 to 16.5)	15.9 (13.7 to 18.3)	15.7 (13.5 to 18.1)	12.5 (10.5 to 14.7)
Infection	643	14.1 (12.0 to 16.4)	6.8 (5.4 to 8.5)	13.6 (11.6 to 15.9)	20.1 (17.6 to 22.9)
Trauma	821	14.0 (12.0 to 16.3)	16.6 (14.4 to 19.1)	18.5 (16.1 to 21.1)	20.5 (18.0 to 23.3)
Illicit drug use	55	1.7 (1.0 to 2.6)	0.8 (0.3 to 1.6)	0.8 (0.3 to 1.5)	1.4 (0.8 to 2.2)
Suicide	477	9.4 (7.8 to 11.4)	10.2 (8.4 to 12.2)	11.6 (9.7 to 13.7)	9.3 (7.7 to 11.3)
SUDIC	1,881	36.8 (33.4 to 40.4)	35.8 (33.4 to 40.4)	42.3 (38.7 to 46.2)	45.1 (41.3 to 49.1)
Underlying disease	4,056	92.7 (87.3 to 98.4)	69.2 (64.5 to 74.1)	84.7 (79.5 to 90.1)	97.8 (92.2 to 103.6)
**Incidence rate ratio (IRR) of death compared to 2019−2020**					
All deaths	12,828	1 (Ref)	0.88 (0.84 to 0.93)	1.01 (0.96 to 1.06)	1.08 (1.03 to 1.13)
*Death by cause*					
Malignancy	1,041	1 (Ref)	1.02 (0.86 to 1.21)	0.97 (0.81 to 1.15)	1.03 (0.87 to 1.23)
Preterm birth	2,860	1 (Ref)	0.95 (0.85 to 1.05)	1.08 (0.98 to 1.20)	1.06 (0.96 to 1.18)
Intrapartum event	686	1 (Ref)	1.12 (0.91 to 1.38)	1.11 (0.90 to 1.37)	0.88 (0.71 to 1.10)
Infection	643	1 (Ref)	0.48 (0.37 to 0.63)	0.96 (0.78 to 1.20)	1.43 (1.17 to 1.74)
Trauma	821	1 (Ref)	1.19 (0.97 to 1.46)	1.32 (1.08 to 1.62)	1.47 (1.20 to 1.79)
Illicit drug use	55	1 (Ref)	0.50 (0.23 to 1.07)	0.45 (0.20 to 0.99)	0.80 (0.41 to 1.54)
Suicide	477	1 (Ref)	1.08 (0.84 to 1.40)	1.23 (0.95 to 1.57)	0.99 (0.76 to 1.29)
SUDIC	1,881	1 (Ref)	0.97 (0.85 to 1.11)	1.15 (1.01 to 1.31)	1.23 (1.08 to 1.39)
Underlying disease	4,056	1 (Ref)	0.75 (0.68 to 0.82)	0.91 (0.84 to 0.99)	1.05 (0.97 to 1.15)
**Absolute Difference in Deaths compared to 2019−20**					
All deaths	12,828	0 (Ref)	−377 (−530 to 224)	31 (−126 to 189)	258 (97 to 419)
*Death by cause*					
Malignancy	1,041	0 (Ref)	5 (−40 to 50)	−9 (−53 to 35)	9 (−36 to 54)
Preterm birth	2,860	0 (Ref)	−38 (−110 to 34)	58 (−17 to 133)	44 (−30 to 118)
Intrapartum event	686	0 (Ref)	20 (−17 to 57)	18 (−19 to 55)	−20 (−55 to 15)
Infection	643	0 (Ref)	−86 (−117 to −55)	−6 (−41 to 29)	71 (32 to 110)
Trauma	821	0 (Ref)	31 (−6 to 68)	53 (15 to 91)	77 (37 to 117)
Illicit drug use	55	0 (Ref)	−10 (−21 to 1)	−11 (−22 to 0)	−4 (−16 to 8)
Suicide	477	0 (Ref)	9 (−21 to 39)	25 (−6 to 56)	−1 (−30 to 28)
SUDIC	1,881	0 (Ref)	−14 (−71 to 43)	65 (5 to 125)	98 (37 to 159)
Underlying disease	4,056	0 (Ref)	−277 (−362 to −191)	−95 (−185 to −5)	60 (−33 to 153)

Numbers are rates per 1,000,000 children per year, Incidence rate ratio (IRR), or excess deaths, all with 95% CI.

**Table 3:** Trends for overall, and categories of deaths, across the whole time period, and split between the two time periods (total population estimate = 11,777,798).

Measure	*N*	Trend over whole period (2019–2023)		Trend over 1st (2019–2021) or 2nd (2021–2023) period		
		IRR (95% CI)	*p*_**trend**_	Period 1 (April 2019–March 2021)	Period 2 (April 2021–March 2023)	Evidence of change in trajectory
All deaths	12,828	1.04 (1.02–1.05)	<0.001	0.96 (0.92–0.99)	1.12 (1.08–1.16)	<0.001
*Death by cause*						
Malignancy	1,041	1.01 (0.95–1.06)	0.831	1.00 (0.89–1.13)	1.01 (0.90–1.13)	0.947
Preterm birth	2,860	1.03 (1.00–1.07)	0.046	1.01 (0.94–1.09)	1.05 (0.98–1.13)	0.562
Intrapartum event	686	0.95 (0.89–1.02)	0.135	1.15 (1.00–1.34)	0.78 (0.68–0.91)	0.004
Infection	643	1.21 (1.13–1.30)	<0.001	0.71 (0.60–0.84)	1.80 (1.57–2.07)	<0.001
Trauma	821	1.12 (1.06–1.20)	<0.001	1.19 (1.03–1.38)	1.07 (0.94–1.21)	0.358
Illicit drug use	55	0.90 (0.71–1.13)	0.363	0.52 (0.31–0.87)	1.58 (0.94–2.67)	0.021
Suicide	477	1.01 (0.93–1.09)	0.849	1.17 (0.97–1.40)	0.88 (0.74–1.04)	0.073
SUDIC	1,881	1.09 (1.04–1.13)	<0.001	1.04 (0.95–1.14)	1.13 (1.04–1.23)	0.281
Underlying disease	4,056	1.04 (1.01–1.07)	0.006	0.85 (0.80–0.90)	1.25 (1.18–1.32)	<0.001

Numbers are Incidence rate ratios (IRRs) (95% CI).

*P*-values derived from Poisson regression, or likelihood ratio test (evidence of change in trajectory).

**Fig 1:**
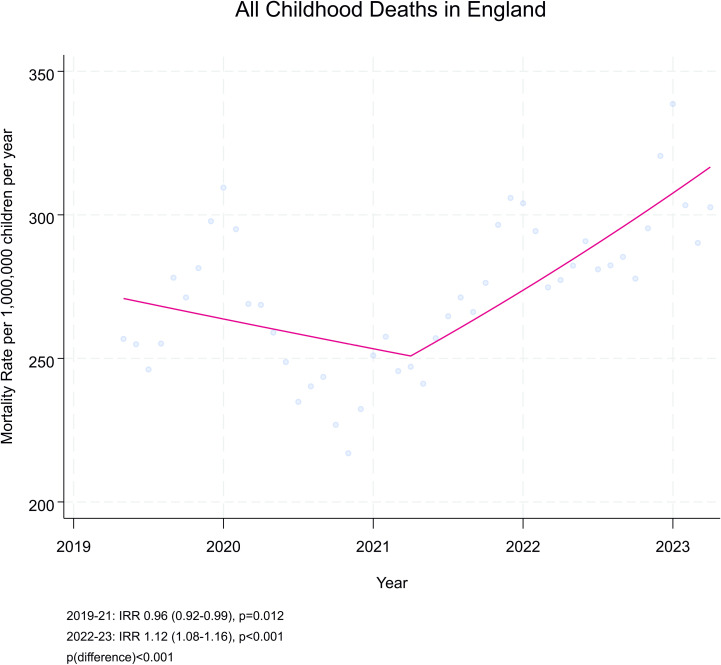
Rate of death (per 1,000,000 person years) per month, and trends of rate across the two study periods (April 2019–March 2022 and April 2021–March 2023). Incidence rate ratios (IRRs) (95%CI). Rates per month smoothed over 5-month period. *P*-values derived from Poisson regression.

**Fig 2:**
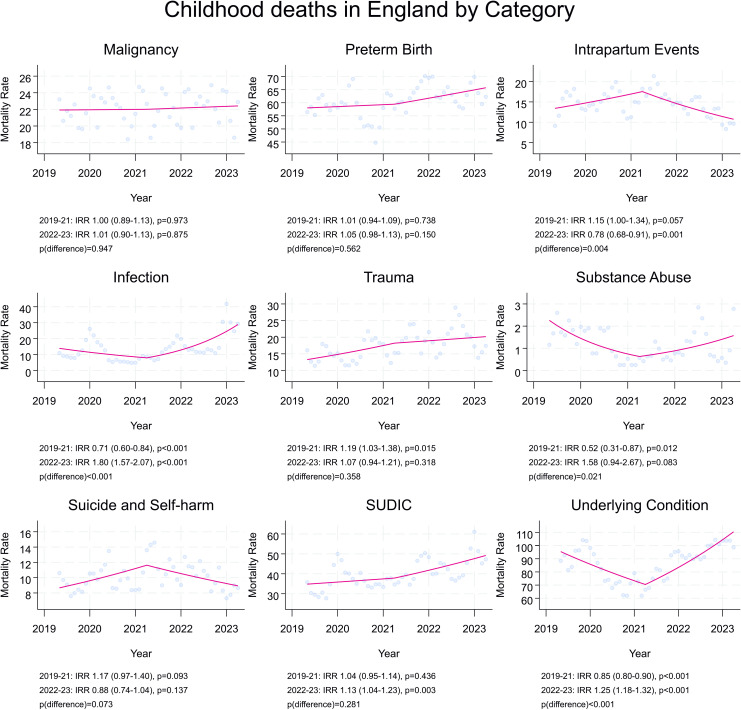
Rate of death per month (per 1,000,000 person years) and trends of rate across the two study periods (April 2019–March 2022 and April 2021–March 2023), split by category of death. Incidence rate ratios (IRRs) (95% CI). Rates per month smoothed over 5-month period. *P*-values derived from Poisson regression.

There was evidence that deaths reduced across Period 1 (IRR 0.96 (95% CI 0.92–0.99)) and then increased in Period 2 (IRR 1.12 (95% CI 1.08–1.16)) (*p*_difference_ < 0.001) (Fig 1 and Table 3). A similar profile was seen for deaths from underlying disease and from infection (Infection, *p* < 0.001; Underlying Disease, *p* < 0.001) (Fig 2). In contrast, for deaths after Intrapartum events, there was an increase in rate across the first period (IRR 1.15 (95% CI 1.00–1.34)), followed by a reduction in rate across the second period (IRR 0.78 (95% CI 0.68–0.91)) (*p* = 0.004). The difference in rates of deaths from suicide in the first and second periods were not statistically significant (Period 1 IRR 1.17 (95% CI 0.97–1.40)) versus Period 2 (IRR 0.88 (95% CI 0.74–1.04), *p*_difference_ = 0.073).”.

Rates of death from preterm birth, trauma and SUDIC, increased across the entire 4-year-study period, with no evidence of a change in trajectory across the two periods (preterm birth, *p* = 0.562; trauma, *p* = 0.358; SUDIC, *p* = 0.281) (Table 3). There was no change in the rate of death from malignancy overall (*p* = 0.831), or any change in trend over the study (*p* = 0.947).

Repeating the analysis, split by child characteristics, suggested that patterns of changing mortality were not modified by sex (*p* = 0.098), area of residence (*p* = 0.176) or region of England (*p* = 0.256) (Table 4). However, the profile did differ by age (*p* < 0.001). For children between 1 and 4 years, the trajectory appeared to change between the two periods (*p* < 0.001), with a reduction in the first period (IRR 0.85 (95% CI 0.76–0.94)), followed by a rise in the second (IRR 1.31 (95% CI 1.19–1.43)). For children below 1 year of age and those between 5 and 15 years, we observed increased rates of deaths in Period 2, and a significant change in the trajectory of death rates between Periods 1 and 2 (Infants, Period 1, IRR 0.97 (95% CI 0.93–1.01), Period 2 (1.05 (95% CI 1.01–1.10)); 5–15 year, Period 1 IRR 0.95 (95% CI 0.88–1.03)), Period 2 IRR 1.23 (95% CI 1.15–1.32))”. In contrast, the rate appeared to increase across both time periods for children in the oldest age group (16 and 17 years old) (IRR 1.08 (95% CI 1.03–1.14)). The pattern of mortality also appeared to differ by ethnicity with children from Asian or Asian British (*p* < 0.001), black or black British (*p* = 0.012) and Other (*p* = 0.003) groups having no evidence of a change in mortality across the first period, and then an increase in the second. For children from white and mixed background, there was a similar increase across both time periods (white CYP (IRR 1.05 (95% CI 1.03–1.07), mixed background (IRR 1.05 (95% CI 0.99–1.12)). For measures of deprivation, there was evidence of a change in the trajectory between periods for the six most deprived deciles; with no change or a decrease, in Period 1, then a rise in Period 2. We observed a general increase in mortality for the least deprived group (Deciles 9/10, IRR 1.06 (95% CI 1.01–1.10), or no evidence of change (Deciles 7/8, IRR 1.02 (95% CI 0.98–1.07)) across the whole 4 years.

**Table 4:** Relative rate of death, per year, over the whole 4 years, and split into the two periods; results stratified by CYP characteristics.

Measure	*N*	Population estimate	Trend over whole period (2019–2023)		Trend over 1st (2019–2021) or 2nd (2021–2023) period			
			IRR (95% CI)	*p*_trend_	Period 1 (April 2019–March 2021)	Period 2 (April 2021–March 2023)	Evidence of change in trajectory	*p*_interaction_
Age of death	12,828							<0.001
<1 years	7,622	580,965	1.01 (0.99–1.03)	0.244	0.97 (0.93–1.01)	1.05 (1.01–1.10	0.041	
1–4 years	1,475	2,498,392	1.07 (1.02–1.12)	0.004	0.85 (0.76–0.94)	1.31 (1.19–1.43)	<0.001	
5–15 years	2,643	7,406,747	1.09 (1.06–1.13)	<0.001	0.95 (0.88–1.03)	1.23 (1.15–1.32)	<0.001	
16–17 years	1,088	1,291,753	1.08 (1.03–1.14)	0.004	1.05 (0.93–1.18)	1.11 (0.99–1.24)	0.592	
Sex	12,774							0.098
Female	5,499	5,741,634	1.02 (1.00–1.05)	0.082	0.97 (0.93–1.02)	1.13 (1.09–1.18)	<0.001	
Male	7,275	6,036,147	1.06 (1.04–1.08)	<0.001	0.94 (0.89–0.99)	1.10 (1.05–1.16)	<0.001	
Area of residence	12,782							0.176
Rural	1,450	1,868,079	0.91 (0.82–1.01)	0.083	0.91 (0.82–1.01)	1.15 (1.05–1.27)	0.010	
Urban (London)	2,187	1,897,050	1.01 (0.97–1.05)	0.657	0.93 (0.86–1.01)	1.08 (1.00–1.17)	0.041	
Urban (not London)	9,145	8,011,752	1.05 (1.03–1.07)	<0.001	0.97 (0.93–1.02)	1.12 (1.08–1.16)	<0.001	
Ethnicity	12,142							<0.001
Asian or British Asian	2,242	1,449,633	1.10 (1.06–1.15)	<0.001	0.93 (0.86–1.01)	1.28 (1.18–1.38)	<0.001	
Black or British black	1,039	671,582	1.12 (1.06–1.18)	<0.001	0.97 (0.85–1.10)	1.27 (1.14–1.42)	0.012	
Mixed	742	801,478	1.05 (0.99–1.12)	0.097	1.04 (0.90–1.20)	1.07 (0.94–1.22)	0.823	
Other^a^	361	312,840	1.13 (1.03–1.24)	0.008	0.84 (0.68–1.04)	1.45 (1.20–1.75)	0.003	
White	7,758	8,541,977	1.05 (1.03–1.07)	<0.001	1.04 (0.99–1.08)	1.06 (1.01–1.10)	0.601	
Region of CDOP	12,828							0.256
East Midlands	1,054	991,086	1.03 (0.98–1.09)	0.201	0.99 (0.88–1.12)	1.07 (0.96–1.20)	0.465	
East of England	1,233	1,330,638	1.02 (0.98–1.08)	0.337	0.93 (0.83–1.04)	1.12 (1.01–1.25)	0.050	
London	2,192	1,898,661	1.01 (0.97–1.04)	0.690	0.93 (0.85–1.01)	1.09 (1.01–1.18)	0.032	
North East	633	525,420	1.11 (1.03–1.18)	0.004	1.04 (0.88–1.22)	1.17 (1.01–1.35)	0.389	
North West	1,903	1,563,550	1.07 (1.03–1.11)	0.001	0.96 (0.88–1.06)	1.17 (1.08–1.27)	0.015	
South East	1,706	1,938,650	1.03 (0.99–1.07)	0.191	0.95 (0.86–1.04)	1.11 (1.01–1.21)	0.069	
South West	947	1,088,112	1.01 (0.96–1.07)	0.644	0.97 (0.85–1.10)	1.05 (0.94–1.19)	0.459	
West Midlands	1,721	1,293,685	1.06 (1.02–1.11)	0.005	0.97 (0.88–1.07)	1.15 (1.05–1.25)	0.002	
Yorkshire and the Humber	1,439	1,147,996	1.07 (1.02–1.12)	0.003	0.97 (0.88–1.08)	1.16 (1.06–1.26)	0.052	
Deprivation decile	12,753							<0.001
1/2 (most deprived)	4,377	2,175,835	1.06 (1.04–1.09)	<0.001	0.95 (0.89–1.01)	1.18 (1.12–1.25)	<0.001	
3/4	2,904	2,141,871	1.04 (1.01–1.08)	0.014	0.97 (0.90–1.04)	1.11 (1.04–1.19)	0.002	
5/6	2,236	2,186,422	1.00 (0.97–1.04)	0.905	0.92 (0.85–1.00)	1.08 (1.00–1.17)	0.033	
7/8	1,760	2,408,474	1.02 (0.98–1.07)	0.274	0.97 (0.88–1.07)	1.07 (0.98–1.17)	0.233	
9/10 (least deprived)	1,476	2,865,174	1.06 (1.01–1.10)	0.014	1.05 (0.95–1.16)	1.07 (0.97–1.17)	0.832	

^a^Other ethnic group (Arab, any other ethnic group).

Incidence rate ratios (IRRs) (95% CI). Rates per month smoothed over 5-month period. *P*-values derived from Poisson regression, or likelihood ratio test (*p*_interaction_ and for evidence of change in trajectory).

**Fig 3:**
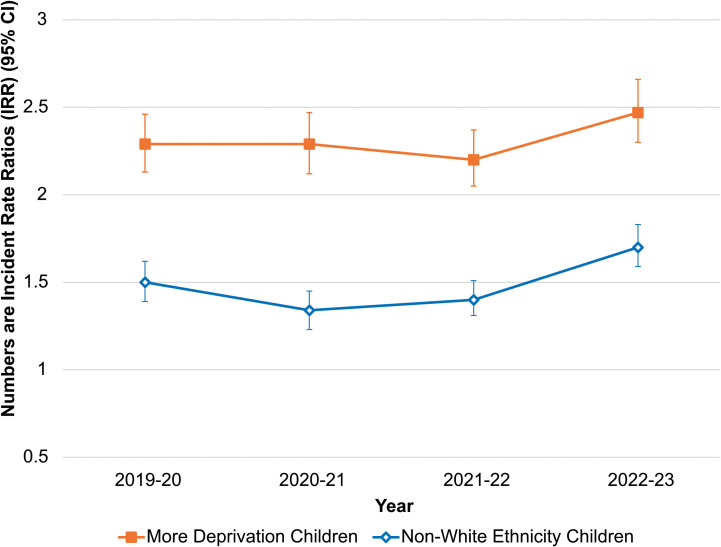
Incidence rate ratios (IRR) with error bars showing 95% CI for living in the more deprived areas of England (compared to the less deprived) areas, and children of non-white ethnicities (compared to those from white background); by year. Incidence rate ratios (IRRs) show relative mortality of CYP in the more deprived 5 quintiles of England with those in the less deprived 5, and the relative mortality of CYP of non-white ethnicities compared to those from white backgrounds.

The relative rate of deaths of children living in the more deprived half of England compared with the less deprived half remained similar across the first two years of this study (*p*_trend_ = 0.526) (Fig 3 and S2 Table). Overall, while there was not statistically significant evidence that inequalities across deprivation levels increased in the second half of the study (*p*_trend_ = 0.093), the highest RR was seen in the final year (2022−2023; IRR 2.47 (95% CI 2.30–2.66)). The relative rate of dying in children from non-white backgrounds, compared to white children, reduced over the first period (*p*_trend_ = 0.021), but then increased (*p*_trend_ < 0.001), to, again, the highest RR seen across the 4 years (2022−2023; IRR 1.70 (95% CI 1.59–1.83). Comparable (absolute) mortality rates are show in S8 Fig.

Stratifying the analysis for the categories of death split by the age category gave compatible analyses (S3 Table) to the main results (Table 3) for malignancy (*p* = 0.497), preterm birth (*p* = 0.691), intrapartum events (*p* = 0.884), illicit drug use (*p* = 0.938), suicide (*p* = 0.820) and SUDIC (*p* = 0.382). The trends of deaths by infection varied by age (*p* = 0.005), with an increase in Period 2 and a significant change in trajectory between the two periods, for all groups below 16/17 years, where there was evidence of a gradual increase across all 4 years (IRR 1.30 (95% CI 1.00–1.70)). Death by trauma also appeared to vary by age, with infant deaths increasing in Period 1 (IRR 1.54 (95% CI 1.01–2.35)) and then decreasing in Period 2 (IRR 0.66 (95% CI 0.44–0.99)). For children between 1 and 4 years, there was little to suggest any change in mortality across study, for children between 5 and 15 years there was no change across Period 1 (IRR 1.06 (95% CI 0.83–1.35)) followed by an increase across Period 2 (IRR 1.25 (95% CI 1.01–1.56)). For children who died between 16 and 17 years old, there was likely an increase across the whole 4 years (IRR 1.20 (95% CI 1.08–1.34)). While the profile for deaths from Underlying Disease did vary by age groups (*p* < 0.001), most age categories showed a reduction in Period 1, followed by an increase in Period 2; although the reduction in 16–17-year-old CYP in Period 1 did not reach statistical significance (Period 1, IRR 0.83 (95% CI 0.63–1.09); Period 2, 1.38 (95% CI 1.08–1.76)). After removing the deaths of children born at 23 and 24 weeks of gestation (S4 Table), all results were compatible with the main analysis, except for preterm deaths, where the evidence for a change across the 4 years did not reach conventional levels of statistical significance (IRR 1.01 (95% CI 0.97–1.05), *p* = 0.649).

Over the 3 years from the start of the national lockdowns (April 2020 to March 2023), there were similar numbers of deaths overall (as predicted by the observed rate seen in 2019−2020: −88 (95% CI −473 to –297)), but more deaths from trauma (161 (95% CI 70–252)) and SUDIC (149 (95% CI 6–292)) and fewer from underlying disease (−312 (95% CI −534 to −90)) (S5 Table).

## Discussion

Overall, child mortality in England after the national lockdowns was higher than during, or preceding it; and this profile is seen across a number of causes of death; including Infections. In contrast some causes of death (notably preterm birth, SUDIC, and trauma) have continued to increase over the 4 years of the study period. Deaths after Intrapartum events may have increased during the national lockdowns, but rates were reducing back to similar baseline levels. While mortality in the most deprived areas, and in children of non-white ethnicities, did not drop during the lockdowns, they demonstrated significant increases in the post-lockdown period, and for one age group (children aged 16 and 17 years) there was a steady increase in mortality across the 4 years.

Overall, the number of deaths of CYP appears to be increasing, and was, across Period 2 above the level seen pre-pandemic [4,5,16], with much of the apparent reduction in mortality likely due to fewer deaths during the national lockdown period. However, four patterns, across the 4 years, appear to exist. In this work, two categories of death appeared to underpin this profile: deaths from infection and underlying disease. Both metrics may be driven by a reduction in circulating pathogens [17], and mediated through the wider public health initiatives [18]. Deaths by illicit drug use fell during the national lockdowns, followed by a statistically significant change in trajectory in Period 2. Without clear data before 2019 this is difficult to interpret, but appears in contrast to some data suggesting increasing illicit drug use during lockdowns in other countries, potentially related to mental health concerns [19].

Two further profiles of mortality were seen in this work. For intrapartum events we saw an increase in the number of deaths during the national lockdowns, and then a subsequent reduction. Differences in the rate of deaths by suicide during and after the two periods did not reach conventional levels of statistical significance. For both of these categories, a lack of detailed data prior to 2019−2020 limits the interpretation, and the trajectory of these deaths, had the pandemic not occurred, is unclear. Suicide in CYP has risen in many other countries over the last 10 years [20], and the results here may represent an existing trend, or an effect of the social restrictions [21]. Perhaps the most surprising findings here were the conditions where, despite COVID, lockdown and broad societal change, little association of the pandemic and the lockdowns was apparent. Apparently indifferent to broad effects on society and healthcare, we saw a general rise across the 4 years for deaths caused by preterm birth, trauma and SUDIC.

The rise in preterm deaths may be due in part to an increasing recognition and registration of ‘livebirth’ in infants born at 22 and 23 weeks of gestation, consistent with other work [22]; NCMD does not receive details of stillborn infants, which limits further interpretation. However, death after preterm birth continues to increase to the highest level since NCMD started collecting data and remains a substantial contributor to the further widening of health inequalities in England [23,24]. Finally, malignancy, a common cause of childhood death, continues to show little, to no, overall change across the time-period studied. Despite concerns related to later diagnosis or initiation of treatment, deaths from malignancy (the 3rd highest category in this work) remains similar to 2019−2020 levels, although we do know that this group was the one of the most vulnerable [2,25]. As with preterm birth, since the underpinning rate of death in this group is high [26] the effect of the pandemic may not have been large enough to be identified.

This work is based on the ongoing data collection from the NCMD, and previous work has shown good validation and coverage [27]. While we had some missing data on some demographics (e.g., ethnicity), data completeness was generally good, and the primary analysis was based on the statutory reporting of deaths, which continued throughout the pandemic. ONS data on deaths in childhood is limited, and covers a slightly different remit to the NCMD, but here we report similar numbers where comparisons are possible (2020–2022 data: ONS deaths 9,063, NCMD deaths 9,419) (S6 Table with details of limitations and caveats). We have used ONS data to derive the population at risk, and this was estimated at a mid-point of the study. Changes may have biased our estimates (e.g., significant, differential migration, during the pandemic), and consequently absolute rates in particular should be interpreted with caution. However, the denominator population came from the 2021 National Census and numbers of deaths are relatively small; making changes in the denominators unlikely to change the conclusions here. Fortunately, child death in England is rare, particularly for causes such as suicide and illicit drug use deaths, which may further limit interpretation; particularly in the stratified analyses. In particular, we have presented the linear trends, smoother monthly rates, and the possible impact of the pandemic and national lockdowns for all categories of death, for the reader to interpret themselves. In addition, the aim of this work was not to investigate any step change in mortality, and single events (e.g., a heat wave and drowning deaths), perhaps different for each category of death, may have affected the predicted trends reported.

Finally, due to the statutory nature of the CDOP process, we anticipate being aware of all deaths in England by the time this data was analysed (for this time period); however while the main outcome (of child death) is unaffected by the coding process, the results from each category of death in this work is provisional on data available to the team at the notification, and may change as further information is provided during the subsequent CDOP investigations and report. In particular the conclusions and results from SUDIC are the most likely to be modified by this process, and should be interpreted with caution.

Overall, while the number of deaths in CYP may have drop during the lockdown period, any overall reduction in mortality appears to have been short lived. Underpinning mechanisms may relate to circulating pathogens returning to pre-pandemic levels, cessation of enhanced health-related behaviour changes (e.g., enhanced hand washing) [28] or withdrawal of wider, state-based enhanced social support, which benefited the most socially vulnerable families. As well as medically vulnerable children, underlying mortality is higher in those from more deprived areas [29], and for some minority ethnic groups [23,30]. While mortality in these groups did not drop during the lockdowns, there were significant increases in the post-lockdown period; leading to a higher relative rate for death in non-white ethnic groups (compared to white CYP) after the lockdown than before or during it. The changes seen deaths by intrapartum events also occurred on a background of national initiatives to reduce brain injury, and death, around birth (e.g., the Each Baby Counts program) [31], and while the recent reduction in deaths by intrapartum events back to pre-pandemic levels is welcome, further close monitoring is essential. Strikingly death by trauma is now the 4th most common cause of childhood deaths in England. In a sensitivity analysis there was some evidence of a bigger increase in trauma deaths in infants during the national lockdowns, followed by a fall afterwards. While this may represent increasing in death from non-accidental injury, as postulated by others [32], other causes, such as drownings and road traffic events [33] are included here, and further work investigating the relative causes across this broad range of death is important and underway.

Any mortality gains, seen during the wide-ranging social changes put in place to reduce the spread and impact of COVID-19, have dissipated. Overall death rate, and deaths by Infection and Underlying Disease, reduced across Period 1 and then increased across Period 2, resulting in Child mortality in 2022−2023 in England higher than before the national lockdowns. Two causes of death, trauma and SUDIC, show clear increases in incidence over the 4 years, with only Intrapartum event deaths showing clear evidence of a reducing trend. For all other causes of death, rates are either static, or worsening. In addition, the relative rate of dying for children from non-white backgrounds, compared to white children, is now higher than before or during the lockdowns.

## Supporting information

S1 RECORD Checklist The RECORD statement—checklist of items, extended from the STROBE statement, that should be reported in observational studies using routinely collected health data. (PDF)

S1 Table Number and proportion of child deaths, overall and by categories, reported to NCMD in England between April 2019 and March 2023. *P*-values derived from *χ*^2^ testing.
(PDF)

S2 Table Incidence rate ratios (IRRs) for death, for each year of the study, compared by measures of deprivation and ethnicity. (a) Other ethnic group (Arab, any other ethnic group). Numbers are IRRs (95% CI). *P*-values derived from Poisson regression.
(PDF)

S3 Table Trends for categories of deaths, across the whole time period, and split between the two time periods, and the age at death. Numbers are incidence rate ratios (IRRs) (95% CI). *P*-values derived from Poisson regression (*p*_trend_), or likelihood ratio test (*p*_interaction_).
(PDF)

S4 Table Trends for overall, and categories of deaths, across the whole time period, and split between the two time periods (Restricted to children born at, or over 24 weeks of gestation). Numbers are incidence rate ratios (IRRs) (95% CI). *P*-values derived from Poisson regression (*p*_trend_).
(PDF)

S5 Table Excess deaths, per year, compared to the pre-lockdown period. Number are number of excess deaths seen (95% CI).
(PDF)

S6 Table Comparison of NCMD and Office of National Statistics (ONS) Data. N.B. Comparison data here is limited to January 2020 to December 2022, for CYP dying before their 16th birthday. Further limitations included differences in included population due to differences in gestational age measures, the handling of non-resident deaths, and deaths abroad.
(PDF)

S1 Fig Rate of death per month (per 1,000,000 person years) and trends of rate across study period, split by ethnicity. Incidence rate ratios (IRRs) (95% CI). Rates per month smoothed over 5-month period. *P*-values derived from Poisson regression, or likelihood ratio test (*p*_interactions_).
(PDF)

S2 Fig Rate of death per month (per 1,000,000 person years) and trends of rate across study period, split by deprivation. Incidence rate ratios (IRRs) (95% CI). Rates per month smoothed over 5-month period. *P*-values derived from Poisson regression, or likelihood ratio test (*p*_interactions_).
(PDF)

S3 Fig Rate of death per month (per 1,000,000 person years) and trends of rate across study period, split by sex. Incidence rate ratios (IRRs) (95% CI). Rates per month smoothed over 5-month period. *P*-values derived from Poisson regression, or likelihood ratio test (*p*_interactions_).
(PDF)

S4 Fig Rate of death per month (per 1,000,000 person years) and trends of rate across study period, split by area. Incidence rate ratios (IRRs) (95% CI). Rates per month smoothed over 5-month period. *P*-values derived from Poisson regression, or likelihood ratio test (*p*_interactions_).
(PDF)

S5 Fig Rate of death per month (per 1,000,000 person years) and trends of rate across study period, split by region of England. Incidence rate ratios (IRRs) (95% CI). Rates per month smoothed over 5-month period. *P*-values derived from Poisson regression, or likelihood ratio test (*p*_interactions_).
(PDF)

S6 Fig Rate of death per month (per 1,000,000 person years) and trends of rate across study period, split by age. Incidence rate ratios (IRRs) (95% CI). Rates per month smoothed over 5-month period. *P*-values derived from Poisson regression, or likelihood ratio test (*p*_interactions_).
(PDF)

S7 Fig Histogram of residuals (the difference between the actual value of a variable and the value predicted by a regression model) of the primary analysis model, of all-cause mortality across the 4-year period. (PDF)

S8 Fig Absolute rate of death by year, split by ethnicity and local deprivation. Rate of death is per 1,000,000 children per year. Error bars show 95% CI.
(PDF)

## Abbreviations

CDOPs: Child Death Overview Panels
CYP: children and young people
GDPR: General Data Protection Regulation
IMD: index of multiple deprivation
IRR: incidence rate ratio
NCMD: National Child Mortality Database
ONS: Office of National Statistics
RECORD: Reporting of Studies Conducted using Observational Routinely-Collected Data
SUDIC: sudden unexpected deaths in infancy and childhood

## References

[1] 14.9 Million excess deaths associated with the COVID-19 pandemic in 2020 and 2021. World Health Organisation; 2022 [cited 17/10/2024]. Available from https://www.who.int/news/item/05-05-2022-14.9-million-excess-deaths-were-associated-with-the-covid-19-pandemic-in-2020-and-2021

[2] Odd D, Stoianova S, Williams T, Thursby-Pelham A, Ladhani S, Oligbu G, et al. Deaths in children and young people in England following SARS-CoV-2 infection during the first two years of the pandemic: a national study using linked mandatory child death reporting data. Research Square. 2023. doi: 10.21203/rs.3.rs-3782971/v1

[3] Flaxman S, Whittaker C, Semenova E, Rashid T, Parks RM, Blenkinsop A, et al. Assessment of COVID-19 as the underlying cause of death among children and young people aged 0 to 19 Years in the US. JAMA Netw Open. 2023;6(1):e2253590. Epub 2023 Jan 03. doi: 10.1001/jamanetworkopen.2022.53590 ; PMCID: PMC9887489 36716029 PMC9887489

[4] Odd D, Stoianova S, Williams T, Fleming P, Luyt K. Child mortality in England during the first year of the COVID-19 pandemic. Arch Dis Child. 2022;107(3):e22. Epub 2021 Dec 06. doi: 10.1136/archdischild-2021-323370 ; PMCID: PMC8662663 34872905 PMC8662663

[5] Odd D, Stoianova S, Williams T, Sleap V, Blair P, Fleming P, et al. Child mortality in England during the COVID-19 pandemic. Arch Dis Child. 2022;107(1):14–20. Epub 2021 Jun 21. doi: 10.1136/archdischild-2020-320899 ; PMCID: PMC8219479 34911683 PMC8219479

[6] Odd D, Stoianova S, Williams T, Fleming P, Luyt K. Child mortality in England during the first 2 years of the COVID-19 pandemic. JAMA Netw Open. 2023;6(1):e2249191. Epub 2023 Jan 03. doi: 10.1001/jamanetworkopen.2022.49191 36622676 PMC9857017

[7] Odd D, Stoianova S, Sleap V, Williams T, Cook N, McGeehan L, et al. Child mortality and social deprivation. National Child Mortality Database (UK); 2021 [cited 17/10/2024]. Available from: https://www.ncmd.info/wp-content/uploads/2021/05/NCMD-Child-Mortality-and-Social-Deprivation-report_20210513.pdf

[8] COVID-19 response—spring 2021 (roadmap). Cabinet Office (UK); 2021 [cited 17/10/2024]. Available from: https://www.gov.uk/government/publications/covid-19-response-spring-2021

[9] Odd D, Williams T, Appleby L, Gunnell D, Luyt K. Child suicide rates during the COVID-19 pandemic in England. J Affect Disord Rep. 2021;6:100273. Epub 2021 Nov 20. doi: 10.1016/j.jadr.2021.100273 ; PMCID: PMC8604801 34841386 PMC8604801

[10] Child death review: statutory and operational guidance (England). London, UK: HM Government; 2018 [cited 17/10/2024]. Available from: https://assets.publishing.service.gov.uk/media/637f759bd3bf7f154876adbd/child-death-review-statutory-and-operational-guidance-england.pdf

[11] Census 2021: Office for National Statistics (ONS); 2022 [cited 17/10/2024]. Available from: https://www.ons.gov.uk/census

[12] Sidebotham P, Fox J, Horwath J, Powell C, Shahid P. Preventing childhood deaths. Department for Children, Schools and Families (UK); 2008.

[13] Area classifications in Great Britain. Office for National Statistics; 2021 [cited 17/10/2024]. Available from: https://www.ons.gov.uk/methodology/geography/geographicalproducts/areaclassifications

[14] McLennan D, Noble S, Noble M, Plunkett E, Wright G, Gutacker N. The English indices of deprivation 2019: technical report. Ministry of Housing, Communities and Local Government; 2019.

[15] Working together to safeguard children. 2018 [cited 17/10/2024]. Available from: https://consult.education.gov.uk/child-protection-safeguarding-and-family-law/working-together-to-safeguard-children-revisions-t/supporting_documents/Working%20Together%20to%20Safeguard%20Children.pdf

[16] Williams T, Sleap V, Stoianova S, Rossouw G, Cook N, Odd D, et al. NCMD second annual report. National Child Mortality Database (UK); 2021 [cited 17/10/2024]. Available from: https://www.ncmd.info/publications/2nd-annual-report

[17] Infection related deaths of children and young people in England. National Child Mortality Database (NCMD); 2023 [cited 17/10/2024]. Available from https://www.ncmd.info/publications/child-death-infection/

[18] COVID-19: guidance for people whose immune system means they are at higher risk. UK Health Security Agency; 2021 [cited 17/10/2024]. Available from: https://www.gov.uk/government/publications/covid-19-guidance-for-people-whose-immune-system-means-they-are-at-higher-risk/covid-19-guidance-for-people-whose-immune-system-means-they-are-at-higher-risk

[19] Shoshani A, Kor A, Farbstein-Yavin S, Gvion Y. Risk and protective factors for substance use and media addictive behaviors in adolescents during the COVID-19 pandemic. J Adolesc. 2024;96(4):746–59. Epub 2024 Jan 29. doi: 10.1002/jad.12295 . 38284471

[20] Padmanathan P, Bould H, Winstone L, Moran P, Gunnell D. Social media use, economic recession and income inequality in relation to trends in youth suicide in high-income countries: a time trends analysis. J Affect Disord. 2020;275:58–65. Epub 2020 May 22. doi: 10.1016/j.jad.2020.05.057 ; PMCID: PMC7397515 32658824 PMC7397515

[21] Gunnell D, Appleby L, Arensman E, Hawton K, John A, Kapur N, et al. Suicide risk and prevention during the COVID-19 pandemic. Lancet Psychiatry. 2020;7(6):468–71. doi: 10.1016/S2215-0366(20)30171-1 32330430 PMC7173821

[22] Davis PJ, Fenton AC, Stutchfield CJ, Draper ES. Rising infant mortality rates in England and Wales-we need to understand gestation specific mortality. BMJ. 2018;361:k1936. doi: 10.1136/bmj.k1936 29739766

[23] Odd DE, Stoianova S, Williams T, Odd D, Edi-Osagie N, McClymont C, et al. Race and ethnicity, deprivation, and infant mortality in England, 2019-2022. JAMA Netw Open. 2024;7(2):e2355403. Epub 2024 Feb 12. doi: 10.1001/jamanetworkopen.2023.55403 ; PMCID: PMC10862146 38345821 PMC10862146

[24] Odd D, Williams T, Stoianova S, Rossouw G, Fleming P, Luyt K. Newborn health and child mortality across England. JAMA Netw Open. 2023;6(10):e2338055. Epub 2023 Oct 02. doi: 10.1001/jamanetworkopen.2023.38055 ; PMCID: PMC10582783 37847501 PMC10582783

[25] Wilde H, Tomlinson C, Mateen BA, Selby D, Kanthimathinathan HK, Ramnarayan P, et al. Hospital admissions linked to SARS-CoV-2 infection in children and adolescents: cohort study of 3.2 million first ascertained infections in England. BMJ. 2023;382:e073639. Epub 2023 Jul 05. doi: 10.1136/bmj-2022-073639 ; PMCID: PMC10318942 37407076 PMC10318942

[26] Odd D, WIlliams T, Stoianova S, Sleap V, Glover N, Rossouw G, et al. The Contribution of Newborn Health to Child Mortality across England. National Child Mortality Database (UK); 2022 [cited 17/10/2024]. Available from: https://www.ncmd.info/wp-content/uploads/2022/07/Perinatal-FINAL.pdf

[27] Battersby C, Statnikov Y, Santhakumaran S, Gray D, Modi N, Costeloe K, et al. The United Kingdom National Neonatal Research Database: a validation study. PLoS One. 2018;13(8):e0201815. doi: 10.1371/journal.pone.0201815 30114277 PMC6095506

[28] Sansone V, Miraglia Del Giudice G, Della Polla G, Angelillo IF. Impact of the COVID-19 pandemic on behavioral changes in healthcare workers in Italy. Front Public Health. 2024;12:1335953. Epub 2024 Feb 22. doi: 10.3389/fpubh.2024.1335953 ; PMCID: PMC10879601 38384871 PMC10879601

[29] Odd D, Stoianova S, Williams T, Odd D, Kurinczuk JJ, Wolfe I, et al. What is the relationship between deprivation, modifiable factors and childhood deaths: a cohort study using the English National Child Mortality Database. BMJ Open. 2022;12(12):e066214. Epub 2022 Dec 09. doi: 10.1136/bmjopen-2022-066214 ; PMCID: PMC9743372 36600341 PMC9743372

[30] Freemantle N, Wood J, Griffin C, Gill P, Calvert MJ, Shankar A, et al. What factors predict differences in infant and perinatal mortality in primary care trusts in England? A prognostic model. BMJ. 2009;339:b2892. Epub 2009 Aug 04. doi: 10.1136/bmj.b2892 ; PMCID: PMC2721034 19654185 PMC2721034

[31] Each Baby Counts: 2019 Progress Report. Royal College of Obstetricians and Gynaecologists. 2020 [cited 17/10/2024]. Report. Available from: https://www.rcog.org.uk/media/qhzlelnc/each-baby-counts-2019-progress-report.pdf

[32] Carsley S, Thomas S, Oei T, Smith B, Harrington D, Pike I, et al. Child abuse and neglect during the COVID-19 pandemic: an umbrella review. Child Abuse Negl. 2024;149:106645. Epub 2024 Jan 19. doi: 10.1016/j.chiabu.2024.106645 38241804

[33] Supramaniam P, Junus S, Hashim L, Chiew SC, Devesahayam PR. Lost years, mortality burden: the impact of COVID-19 pandemic on premature death due to road traffic accidents in a northern state in Malaysia. BMC Public Health. 2024;24(1):1520. Epub 2024 Jun 06. doi: 10.1186/s12889-024-19027-2 ; PMCID: PMC11155150 38844906 PMC11155150

