# Supplementary material for: Child mortality in England after national lockdowns for COVID-19: An analysis of childhood deaths, 2019–2023
Source: PLoS Med. 2025 Jan 23;22(1):e1004417. doi: 10.1371/journal.pmed.1004417 (PMC11756792; doi:10.1371/journal.pmed.1004417)
Supplement: S1 Table — P-values derived from χ2 testing. (PDF) [file pmed.1004417.s002.pdf]

**S1 Table. Number and proportion of child deaths, overall and by categories, reported to NCMD in England between April 2019 and March 2023.**

| Measure | N | Child deaths reported – Number (Percent) | | | | P ($Chi^2$) |
|---|---|---|---|---|---|---|
| | | 2019/2020 | 2020/2021 | 2021/2022 | 2022/2023 | |
| All Deaths | 12,828 | 3229 | 2852 | 3260 | 3487 | - |
| Death by Cause | 12,520 | | | | | <0.001 |
| Malignancy | 1041 | 259 (22.5%) | 264 (9.6%) | 250 (7.8%) | 268 (7.8%) | |
| Preterm Birth | 743 | 699 (22.5%) | 661 (24.0%) | 757 (23.6%) | 743 (21.6%) | |
| Intrapartum Event | 686 | 167 (5.4%) | 187 (6.8%) | 185 (5.8%) | 147 (4.3%) | |
| Infection | 643 | 166 (5.3%) | 80 (2.9%) | 160 (5.0%) | 237 (6.9%) | |
| Trauma | 821 | 165 (5.3%) | 196 (7.1%) | 218 (6.8%) | 242 (7.0%) | |
| Substance Abuse | 55 | 20 (0.6%) | 10 (0.4%) | 9 (0.3%) | 16 (0.5%) | |
| Suicide | 477 | 111 (3.6%) | 120 (4.4%) | 136 (4.2%) | 110 (3.2%) | |
| SUDIC | 1881 | 433 (13.9%) | 419 (15.2%) | 498 (15.5%) | 531 (15.4%) | |
| Underlying Disease | 4056 | 1092 (35.1%) | 815 (29.6%) | 997 (31.1%) | 1152 (33.4%) | |

P values derived from $Chi^2$ testing
