# Supplementary material for: Child mortality in England after national lockdowns for COVID-19: An analysis of childhood deaths, 2019–2023
Source: PLoS Med. 2025 Jan 23;22(1):e1004417. doi: 10.1371/journal.pmed.1004417 (PMC11756792; doi:10.1371/journal.pmed.1004417)
Supplement: S2 Table — (a) Other ethnic group (Arab, any other ethnic group). Numbers are IRRs (95% CI). P-values derived from Poisson regression. (PDF) [file pmed.1004417.s003.pdf]

S2 Table. Incidence-Rate Ratios for death, for each year of the study, compared by measures of deprivation and ethnicity.

Measure	N	Year				Period 1 (April 2019-March 2021)	Period 2 (April 2021-March 2023)
		2019-2020	2020-2021	2021-2022	2022-2023	p_{trend}	p_{trend}
Deprivation							
Least Deprived Areas	4307	1 (Ref)	1 (Ref)	1 (Ref)	1 (Ref)		-
Most deprived areas	8449	2.29 (2.13-2.46)	2.29 (2.12-2.47)	2.20 (2.05-2.37)	2.47 (2.30-2.66)	0.526	0.093
Ethnicity							
White	7758	1 (Ref)	1 (Ref)	1 (Ref)	1 (Ref)		
Any non-White Ethnicity	4384	1.50 (1.39-1.62)	1.34 (1.23-1.45)	1.40 (1.31-1.51)	1.70 (1.59-1.83)	0.021	<0.001
Asian or British Asian	2242	1.72 (1.57-1.90)	1.50 (1.35-1.66)	1.61 (1.46-1.76)	1.95 (1.79-2.13)	0.029	<0.001
Black or British Black	1039	1.60 (1.40-1.83)	1.68 (1.46-1.92)	1.51 (1.32-1.72)	2.02 (1.79-2.27)	0.297	0.002
Mixed	742	1.08 (0.93-1.25)	0.90 (0.76-1.07)	1.07 (0.93-1.23)	1.02 (0.88-1.18)	0.998	0.908
Other[a]	361	1.31 (1.06-1.62)	0.99 (0.77-1.27)	1.12 (0.90-1.39)	1.63 (1.36-1.95)	0.055	0.001

(a) Other ethnic group (Arab, Any other ethnic group)

Numbers are Incident-Rate Ratios (IRR) (95% Confidence Intervals (CI))

P-values derived from Poisson regression
