# Supplementary material for: Child mortality in England after national lockdowns for COVID-19: An analysis of childhood deaths, 2019–2023
Source: PLoS Med. 2025 Jan 23;22(1):e1004417. doi: 10.1371/journal.pmed.1004417 (PMC11756792; doi:10.1371/journal.pmed.1004417)
Supplement: S3 Table — Numbers are incidence rate ratios (IRRs) (95% CI). P-values derived from Poisson regression (ptrend), or likelihood ratio test (pinteraction). (PDF) [file pmed.1004417.s004.pdf]

S3 Table. Trends for categories of deaths, across the whole time period, and split between the two time periods, and the age at death

Measure	N	Trend over whole period (2019-2023)		Trend over 1st (2019-2021) or 2nd (2021-2023) period			
		IRR (95% CI)	p_{trend}	Period 1 (April 2019-March 2021)	Period 2 (April 2021-March 2023)	Evidence of change in trajectory	$p_{interaction}$
Malignancy							0.497
<1 years	64	0.95 (0.77-1.18)	0.650	0.78 (0.48-1.26)	1.15 (0.72-1.84)	0.376	
1-4 years	237	1.04 (0.93-1.16)	0.475	1.04 (0.80-1.35)	1.04 (0.82-1.32)	0.995	
5-15 Years	611	0.98 (0.91-1.05)	0.533	1.00 (0.86-1.17)	0.96 (0.82-1.11)	0.571	
16-17 Years	129	1.10 (0.94-1.28)	0.227	1.07 (0.75-1.53)	1.12 (0.82-1.54)	0.883	
Preterm Birth							0.691
<1 years	2851	1.03 (1.00-1.07)	0.045	1.01 (0.94-1.09)	1.05 (0.98-1.13)	0.568	
1-4 years	NA	NA	NA	NA	NA	NA	
5-15 Years	NA	NA	NA	NA	NA	NA	
16-17 Years	NA	NA	NA	NA	NA	NA	
Intrapartum event							0.884
<1 years	NA	0.95 (0.89-1.02)	0.146	1.16 (1.00-1.34)	0.79 (0.68-0.91)	0.004	
1-4 years	NA	NA	NA	NA	NA	NA	
5-15 Years	NA	NA	NA	NA	NA	NA	
16-17 Years	NA	NA	NA	NA	NA	NA	
Infection							0.005
<1 years	225	1.10 (0.98-1.24)	0.106	0.81 (0.62-1.06)	1.43 (1.12-1.81)	0.016	
1-4 years	167	1.40 (1.21-1.61)	<0.001	0.53 (0.37-0.76)	2.77 (2.08-3.68)	<0.001	
5-15 Years	207	1.22 (1.08-1.38)	0.002	0.68 (0.50-0.92)	1.86 (1.46-2.38)	<0.001	
16-17 Years	44	1.30 (1.00-1.70)	0.050	1.56 (0.78-3.12)	1.15 (0.69-1.93)	0.580	
Trauma							0.034
<1 years	92	1.00 (0.83-1.19)	0.966	1.54 (1.01-2.35)	0.66 (0.44-0.99)	0.022	
1-4 years	190	1.03 (0.91-1.17)	0.608	1.19 (0.89-1.60)	0.91 (0.69-1.18)	0.272	
5-15 Years	285	1.16 (1.04-1.29)	0.005	1.06 (0.83-1.35)	1.25 (1.01-1.56)	0.044	
16-17 Years	254	1.20 (1.08-1.34)	0.001	1.24 (0.95-162)	1.17 (0.94-1.46)	0.809	
Substance Abuse							0.938
<1 years	NA	NA	NA	NA	NA	NA	
1-4 years	NA	NA	NA	NA	NA	NA	
5-15 Years	NA	NA	NA	NA	NA	NA	
16-17 Years	38	0.99 (0.75-1.31)	0.965	0.60 (0.32-1.12)	1.62 (0.88-2.99)	0.085	
Suicide							0.820

<1 years	NA	NA	NA	NA	NA	NA	
1-4 years	NA	NA	NA	NA	NA	NA	
5-15 Years	217	1.09 (0.97-1.23)	0.142	1.34 (1.01-1.77)	0.92 (0.72-1.17)	0.113	
16-17 Years	260	0.94 (0.85-1.05)	0.264	1.06 (0.83-1.34)	0.84 (0.66-1.06)	0.820	
SUDIC							0.382
<1 years	1185	1.06 (1.01-1.12)	0.018	1.01 (0.90-1.14)	1.11 (1.00-1.23)	0.369	
1-4 years	284	1.11 (1.00-1.23)	0.043	0.87 (0.68-1.10)	1.37 (1.11-1.70)	0.027	
5-15 Years	297	1.18 (1.07-1.30)	0.001	1.33 (1.04-1.70)	1.07 (1.32)	0.294	
16-17 Years	115	1.17 (1.00-1.37)	0.052	1.29 (0.87-1.91)	1.09 (0.78-1.51)	0.609	
Underlying Disease							<0.001
<1 years	2338	1.00 (0.97-1.04)	0.795	0.87 (0.81-0.95)	1.14 (1.06-1.23)	<0.001	
1-4 years	549	1.02 (0.94-1.09)	0.669	0.81 (0.69-0.96)	1.25 (1.07-1.46)	0.004	
5-15 Years	952	1.14 (1.08-1.20)	<0.001	0.82 (0.72-0.93)	1.50 (1.33-1.68)	<0.001	
16-17 Years	217	1.09 (0.97-1.22)	0.145	0.83 (0.63-1.09)	1.38 (1.08-1.76)	0.033	

Numbers are Incidence-Rate Ratios (IRR) (95% Confidence Intervals (CI))

P-values derived from Poisson regression (p_{trend}), or Likelihood Ratio Test ($p_{interaction}$)
