# Supplementary material for: Child mortality in England after national lockdowns for COVID-19: An analysis of childhood deaths, 2019–2023
Source: PLoS Med. 2025 Jan 23;22(1):e1004417. doi: 10.1371/journal.pmed.1004417 (PMC11756792; doi:10.1371/journal.pmed.1004417)
Supplement: S4 Table — Numbers are incidence rate ratios (IRRs) (95% CI). P-values derived from Poisson regression (ptrend). (PDF) [file pmed.1004417.s005.pdf]

S4 Table. Trends for overall, and categories of deaths, across the whole time period, and split between the two time periods (Restricted to children born at, or over 24 weeks of gestation)

**Measure**	**N**	**Trend over whole period (2019-2023)**		**Trend over 1st (2019-2021) or 2nd (2021-2023) period**		
		**IRR (95% CI)**	p_{trend}	**Period 1** (April 2019-March 2021)	**Period 2** (April 2021-March 2023)	**Evidence of change in trajectory**
All Deaths	11,578	1.04 (1.02-1.05)	<0.001	0.95 (0.92-0.99)	1.12 (1.08-1.16)	<0.001
Death by Cause						
Malignancy	1040	1.00 (0.95-1.06)	0.865	1.00 (0.89-1.13)	1.01 (0.90-1.13)	0.914
Preterm Birth	1644	1.01 (0.97-1.05)	0.649	0.99 (0.90-1.09)	1.03 (0.94-1.12)	0.709
Intrapartum event	685	0.95 (0.89-1.01)	0.129	1.15 (0.99-1.33)	0.79 (0.68-0.91)	0.004
Infection	639	1.22 (1.14-1.31)	<0.001	0.73 (0.61-0.86)	1.79 (1.56-2.06)	<0.001
Trauma	820	1.13 (1.06-1.20)	<0.001	1.19 (1.03-1.38)	1.07 (0.94-1.21)	0.366
Substance Abuse	55	0.90 (0.71-1.13)	0.363	0.52 (0.31-0.87)	1.58 (0.94-2.67)	0.021
Suicide	477	1.01 (0.93-1.09)	0.849	1.17 (0.97-1.40)	0.88 (0.74-1.04)	0.073
SUDIC	1876	1.09 (1.04-1.13)	<0.001	1.04 (0.94-1.14)	1.13 (1.04-1.23)	0.275
Underlying Disease	4042	1.04 (1.01-1.07)	0.005	0.85 (0.80-0.91)	1.25 (1.18-1.32)	<0.001

Numbers are Incidence-Rate Ratios (IRR) (95% Confidence Intervals (CI))

P-values derived from Poisson regression (p_{trend})
