# Supplementary material for: Child mortality in England after national lockdowns for COVID-19: An analysis of childhood deaths, 2019–2023
Source: PLoS Med. 2025 Jan 23;22(1):e1004417. doi: 10.1371/journal.pmed.1004417 (PMC11756792; doi:10.1371/journal.pmed.1004417)
Supplement: S5 Table — Number are number of excess deaths seen (95% CI). (PDF) [file pmed.1004417.s006.pdf]

**S5 Table. Excess deaths, per year, compared to the pre-lockdown period.**

| Measure | | N | Reference Year | Excess deaths (Number (95% CI)) | | | |
|---|---|---|---|---|---|---|---|
| | | | 2019-2020 | 2020-2021 | 2021-2022 | 2022-2023 | Total 2020-2023 |
| All Deaths | | 12,828 | Ref | -377 (-530 to -224) | 31 (-127 to 189) | 258 (97 to 419) | -88 (-473 to 297) |
| Death by Cause | | | | | | | |
| Malignancy | | 1041 | Ref | 5 (-40 to 50) | -9 (-53 to 35) | 9 (-36 to 54) | 5 (-104 to 114) |
| Preterm Birth | | 2860 | Ref | -38 (-110 to 34) | 58 (-17 to 133) | 44 (-30 to 118) | 64 (-116 to 244) |
| Intrapartum event | | 686 | Ref | 20 (-17 to 57) | 18 (-19 to 55) | -20 (-55 to 15) | 18 (-70 to 106.2) |
| Infection | | 643 | Ref | -86 (-117 to -55) | -6 (-41 to 29) | 71 (32 to 110) | -21 (-108 to 66) |
| Trauma | | 821 | Ref | 31 (-6 to 68) | 53 (15 to 91) | 77 (37 to 117) | 161 (70 to 252) |
| Substance Abuse | | 55 | Ref | -10 (-21 to 1) | -11 (-22 to 0) | -4 (-22 to 8) | -25 (-54 to 4) |
| Suicide | | 477 | Ref | 9 (-21 to 39) | 25 (-6 to 56) | -1 (-30 to 28) | 33 (-39 to 105) |
| SUDIC | | 1881 | Ref | -14 (-71 to 43) | 65 (5 to 125) | 98 (37 to 159) | 149 (6 to 292) |
| Underlying Disease | | 4056 | Ref | -277 (-363 to 191) | -95 (-185 to -5) | 60 (-33 to 153) | -312 (-534 to -90) |

Number are number of excess deaths seen (95% CI).
