# Supplementary material for: Child mortality in England after national lockdowns for COVID-19: An analysis of childhood deaths, 2019–2023
Source: PLoS Med. 2025 Jan 23;22(1):e1004417. doi: 10.1371/journal.pmed.1004417 (PMC11756792; doi:10.1371/journal.pmed.1004417)
Supplement: S6 Table — N.B. Comparison data here is limited to January 2020 to December 2022, for CYP dying before their 16th birthday. Further limitations included differences in included population due to differences in gestational age measures, the handling of non-resident deaths, and deaths abroad. (PDF) [file pmed.1004417.s007.pdf]

S6 Table. Comparison of NCMD and Office of National Statistics Data (ONS)

Calendar Year	NCMD deaths	ONS deaths
2020	2888	2838
Infants (<1 year)	1996	2100
1-4 years	314	245
5-9 years	209	184
10 to 14 years	276	226
15 years	93	83
2021	3111	3010
Infants (<1 year)	2138	2209
1-4 years	342	271
5-9 years	225	202
10 to 14 years	299	241
15 years	107	87
2022	3420	3215
Infants (<1 year)	2232	2240
1-4 years	419	328
5-9 years	291	245
10 to 14 years	362	303
15 years	115	99

N.B. Comparison data here is limited to January 2020 to December 2022, for CYP dying before their 16th birthday. Further limitations included differences in included population due to differences in gestational age measures, the handling of non-resident deaths, and deaths abroad.
