# Supplementary material for: Child mortality in England after national lockdowns for COVID-19: An analysis of childhood deaths, 2019–2023
Source: PLoS Med. 2025 Jan 23;22(1):e1004417. doi: 10.1371/journal.pmed.1004417 (PMC11756792; doi:10.1371/journal.pmed.1004417)
Supplement: S6 Fig — Incidence rate ratios (IRRs) (95% CI). Rates per month smoothed over 5-month period. P-values derived from Poisson regression, or likelihood ratio test (pinteractions). (PDF) [file pmed.1004417.s013.pdf]

S6 Figure. Rate of death per month (per 1,000,000 person years) and trends of rate across study period, split by age

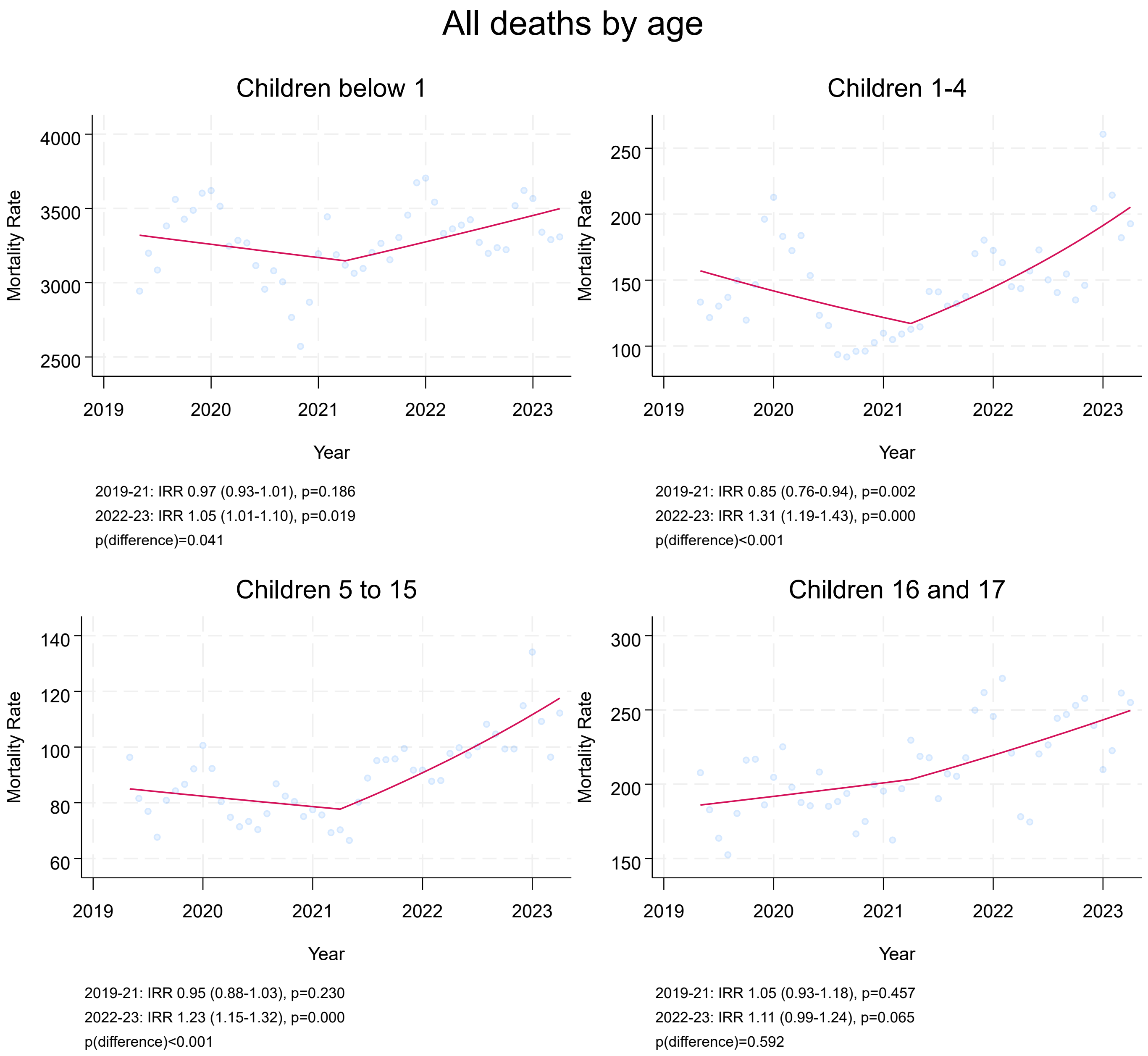

Interaction p<0.001

Incidence-Rate Ratios (IRR) (95% Confidence Intervals (CI)). Rates per month smoothed over 5 month period. P-values derived from Poisson regression, or Likelihood Ratio Test (p for Interactions)
