# Supplementary material for: Child mortality in England after national lockdowns for COVID-19: An analysis of childhood deaths, 2019–2023
Source: PLoS Med. 2025 Jan 23;22(1):e1004417. doi: 10.1371/journal.pmed.1004417 (PMC11756792; doi:10.1371/journal.pmed.1004417)

S7 Figure. Histogram of residuals (the difference between the actual value of a variable and the value predicted by a regression model) of the primary analysis model, of all-cause mortality across the 4 year period.

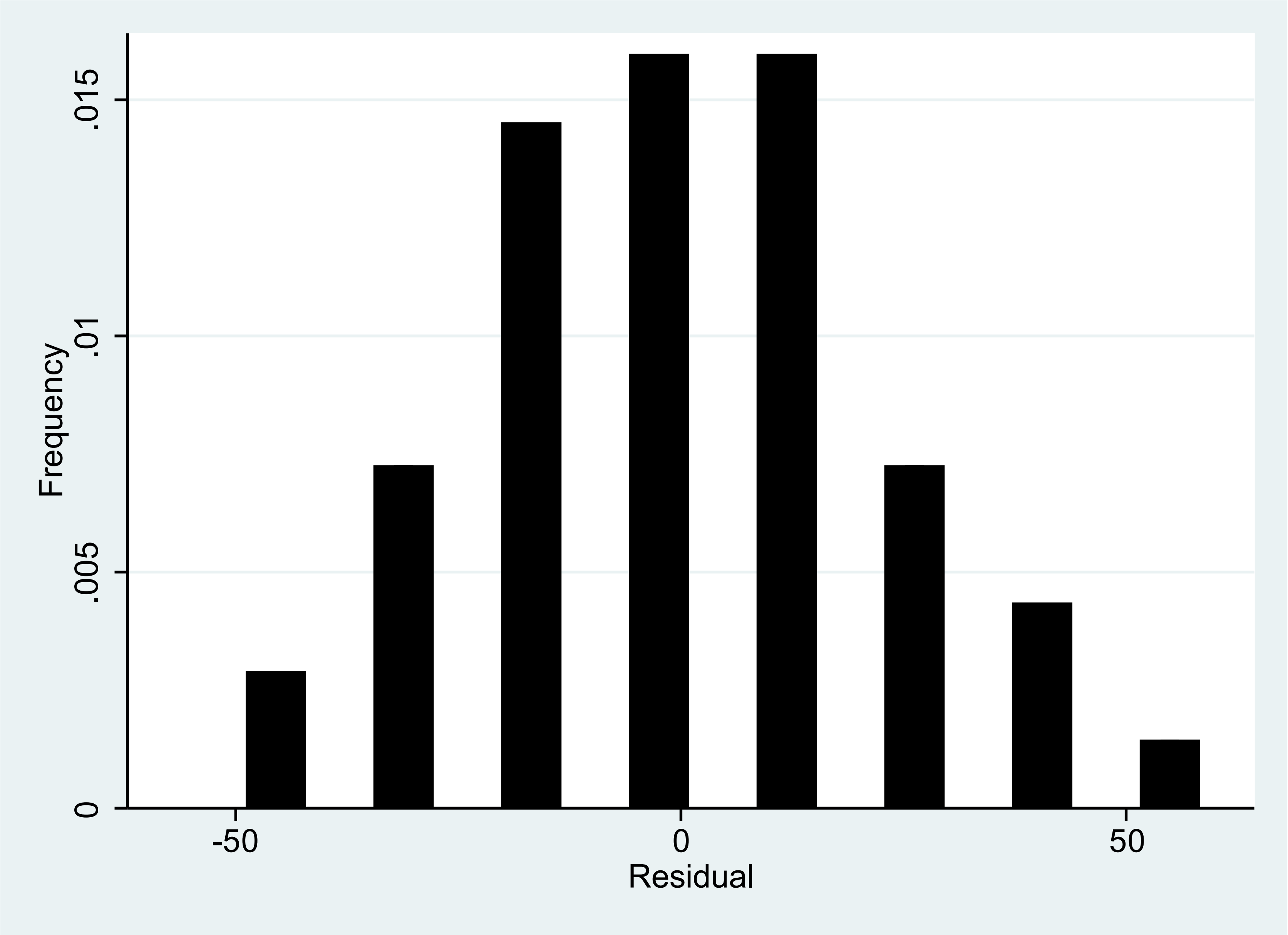

Supplement: S7 Fig — (PDF) [file pmed.1004417.s014.pdf]
