# Supplementary material for: Child mortality in England after national lockdowns for COVID-19: An analysis of childhood deaths, 2019–2023
Source: PLoS Med. 2025 Jan 23;22(1):e1004417. doi: 10.1371/journal.pmed.1004417 (PMC11756792; doi:10.1371/journal.pmed.1004417)
Supplement: S8 Fig — Rate of death is per 1,000,000 children per year. Error bars show 95% CI. (PDF) [file pmed.1004417.s015.pdf]

S8. Figure. Absolute Rate of Death by year, split by ethnicity and local deprivation

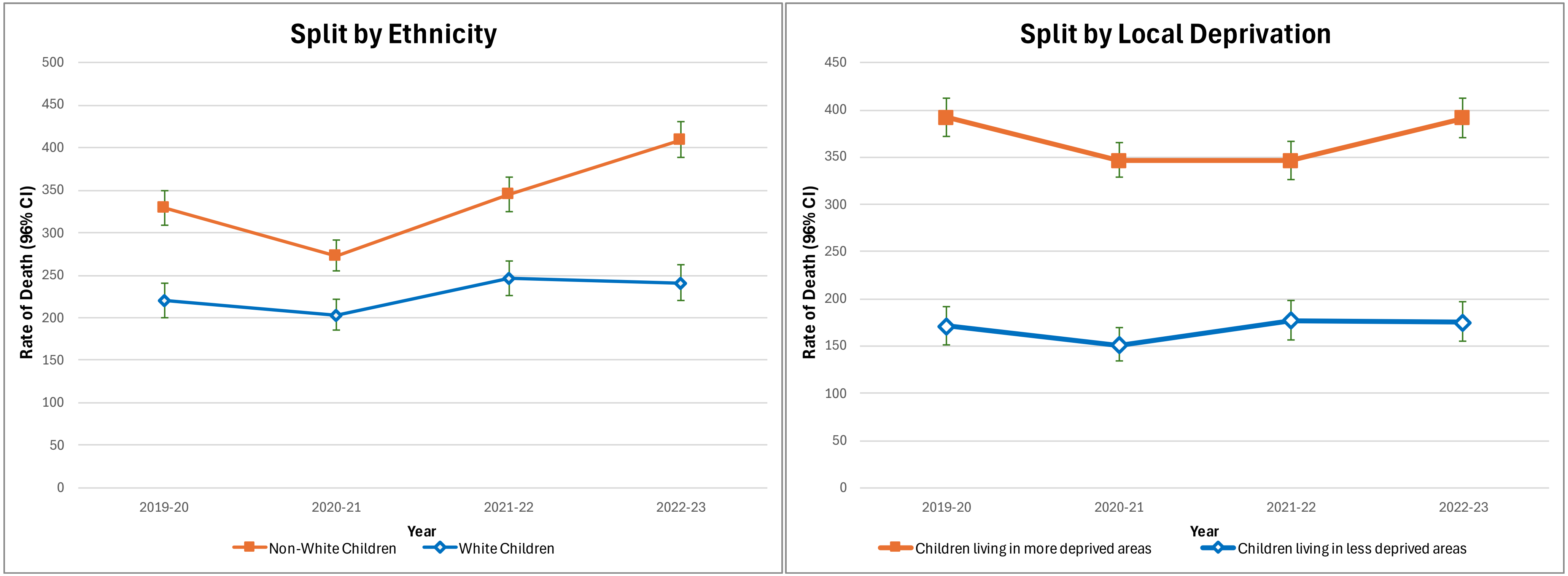

Rate of death is per 1,000,000 children per year. Error bars show 95% Confidence Intervals (CI)
